# Charged Shear-Free Fluids and Complexity in First Integrals

**DOI:** 10.3390/e24050645

**Published:** 2022-05-04

**Authors:** Sfundo C. Gumede, Keshlan S. Govinder, Sunil D. Maharaj

**Affiliations:** 1 Astrophysics Research Centre, School of Mathematics, Statistics and Computer Science, University of KwaZulu-Natal, Private Bag X54001, Durban 4000, South Africa; gumedesc@mut.ac.za (S.C.G.); govinder@ukzn.ac.za (K.S.G.); 2 Department of Mathematical Sciences, Mangosuthu University of Technology, P.O. Box 12363, Jacobs 4026, South Africa

**Keywords:** relativistic fluids, Einstein-Maxwell field equations, first integrals

## Abstract

The equation $y_{xx}=f\left(x\right)y^{2}+g\left(x\right)y^{3}$ is the charged generalization of the Emden-Fowler equation that is crucial in the study of spherically symmetric shear-free spacetimes. This version arises from the Einstein–Maxwell system for a charged shear-free matter distribution. We integrate this equation and find a new first integral. For this solution to exist, two integral equations arise as integrability conditions. The integrability conditions can be transformed to nonlinear differential equations, which give explicit forms for f(x) and g(x) in terms of elementary and special functions. The explicit forms $f\left(x\right)∼\frac{1}{x^{5}}{\left(1-\frac{1}{x}\right)}^{-11/5}$ and $g\left(x\right)∼\frac{1}{x^{6}}{\left(1-\frac{1}{x}\right)}^{-12/5}$ arise as repeated roots of a fourth order polynomial. This is a new solution to the Einstein-Maxwell equations. Our result complements earlier work in neutral and charged matter showing that the complexity of a charged self-gravitating fluid is connected to the existence of a first integral.

## 1. Introduction

The concept of complexity was introduced by Herrera [1] for self-gravitating systems in general relativity. This approach has proved to be useful for studying the behaviour of highly dense stars, neutron stars and radiating stars in strong gravitational fields. Complexity has been studied in spherical systems, cylindrical systems, axial systems and hyperbolic systems by various researchers [2,3,4,5,6,7,8,9,10,11,12,13,14], showing its applicability in a variety of applications. Apart from general relativity, the concept of complexity has been studied in extended theories of gravity including Einstein–Gauss–Bonnet gravity, Lovelock gravity, f(R) gravity and other generalizations [13,15,16,17,18,19,20]. It is important to obtain a deeper insight into the behaviour of relativistic self-gravitating fluids, including dissipative effects. Charged shear-free relativistic fluids have been applied to many stellar systems including radiating stars with the Vaidya geometry describing the external atmosphere. In this study we focus on charged shear-free fluids with spherical symmetry. A new first integral is identified. This suggests a deeper connection between first integrals, charged dissipative distributions and the complexity of self-gravitating relativistic fluids in general. Observe that it is difficult to explicitly find first integrals in practice. There is no algorithm that generates them systematically. Here we show that a new first integral arises, in a simple approach, by adapting a previous method. We multiply Equation (7) by a function to generate a new differential equation, eventually leading to a new first integral. There is no guarantee that this approach will work in general; we find that this simple idea is an effective procedure for a relativistic charged gravitating fluid.

Exact solutions to the Einstein–Maxwell equations are important in relativistic astrophysics and cosmology as they are used to investigate properties of physical phenomena. The Einstein–Maxwell equations may be used to describe charged compact objects with strong electromagnetic effects [21]. There has been substantial research in seeking exact solutions to the Einstein–Maxwell equations. This research include various treatments of Ivanov [22], Srivastava [23], Sharma et al. [24] and Kweyama et al. [21] among others. Assumptions of spherical symmetry in spacetimes and shear-free matter distributions are usually made when seeking exact solutions to the Einstein field equations with uncharged matter. This simplifies the Einstein field equations to the single partial differential equation $y_{xx}=f\left(x\right)y^{2}$ (which can be treated as an ordinary differential equation). Classes of solutions to this ordinary differential equation have been found by Kustaanheimo and Qvist [25], Srivastava [26], Stephani [27], Stephani et al. [28] and Maharaj et al. [29]. Similarly, when seeking exact solutions to the Einstein–Maxwell equations with charged matter, spherical symmetry and the absence of shear is usually assumed. These assumptions simplify the Einstein–Maxwell equations to the single partial differential equation $y_{xx}=f\left(x\right)y^{2}+g\left(x\right)y^{3}.$ This equation consists of an additional term $g\left(x\right)y^{3}$ compared to its uncharged counterpart. This term is due to the presence of the electromagnetic field. Kweyama et al. [21] investigated integrability and found exact solutions to this equation using an approach suggested by Srivastava [26]. Krasinski [30] provides a review of charged solutions with a Friedmann limit. Sussman [31,32] performed a detailed physical analysis of the Einstein–Maxwell equations. The condition of vanishing shear has been applied to different physical applications in cosmology and astrophysics.

Vanishing shear leads to a simplification of the Einstein–Maxwell equations. An important reason to consider the shear-free condition and homogeneous expansion rate is the connection to the analogue of homologous fluids in the classical Newtonian limit. This implies that the shear-free restriction has a meaningful basis in general relativity, and other gravity theories. It should be noted that shear-free fluids may become unstable because of pertubations due to anisotropic effects and dissipative effects. The stability of shear-free configurations, and general dissipative matter in relativistic astrophysics, has been studied in treatments [33,34,35,36,37,38]. As observed in these studies pressure anisotropy and dissipation are effects that should be studied, including the stability of the configuration, as the relativistic fluid evolves from the isotropic state. These quantities play an important role in models of gravitational collapse.

In this paper we investigate the integrability properties and find exact solutions to the charged field equation $y_{xx}=f\left(x\right)y^{2}+g\left(x\right)y^{3}$ using an ad hoc approach adopted in [29]. In Section 2 we show how the Einstein–Maxwell equations reduce to this master equation and briefly discuss the results obtained by Kweyama et al. [21]. We obtain our new first integral in Section 3. This first integral is subject to two integrability conditions which are integral equations. We solve these integral equations in Section 4. We find restrictions on the functions f(x) and g(x) in Section 5. Our results indicate that first integrals are obtainable for charged shear-free fluids extending the result of Gumede et al. [39]. Important technical information, providing detail for the calculations performed, are provided in Appendices Appendix A and Appendix B.

## 2. Charged Shear-Free Fluids

The set of the Einstein–Maxwell equations follow from variation of the Lagrangian:

(1) $$L=\frac{1}{2}\left(R-\frac{1}{4}F_{ab}F^{ab}\right)+L_{m},$$

where *R* is the Ricci scalar, $F_{ab}$ is the electromagnetic field tensor and $L_{m}$ represents the matter source. Variation of the Lagrangian *L* leads to the Einstein–Maxwell equations:

(2a) $$\begin{array}{ccc} R_{ab}-\frac{1}{2}Rg_{ab} & = & \left(\mu +p\right)u_{a}u_{b}+pg_{ab}+2\left({F_{a}}^{c}F_{bc}-\frac{1}{4}g_{ab}F_{cd}F^{cd}\right), \end{array}$$

(2b) $$\begin{array}{ccc} F_{ab;c}+F_{bc;a}+F_{ca;b} & = & 0, \end{array}$$

(2c) $$\begin{array}{ccc} F^{ab}{}_{;b} & = & \frac{1}{2}J^{a}, \end{array}$$

for a perfect fluid source with energy density μ and pressure p. Note that $J^{a}=\sigma u^{a}$, where σ is the proper charge density and $u^{a}$ is a timelike fluid 4-velocity.

We consider a spherical spacetime with the metric:

(3) $$ds^{2}=-e^{2\nu (t,r)}dt^{2}+e^{2\lambda (t,r)}\left[dr^{2}+r^{2}\left(d\theta^{2}+\sin^{2}\theta \phi^{2}\right)\right],$$

for a charged perfect fluid in the comoving and isotropic coordinate system $\left(x^{a}\right)=\left(t,r,\theta ,\phi \right).$ The Einstein field equations for the line element (3), for a shear-free and charged matter distribution, can be written as:

(4a) $$\begin{array}{ccc} \mu & = & 3\frac{{\lambda_{t}}^{2}}{e^{2\nu }}-\frac{1}{e^{2\lambda }}\left(2\lambda_{rr}+\lambda_{r}^{2}+\frac{4\lambda_{r}}{r}\right)-\frac{E^{2}}{r^{4}e^{4\lambda }}, \end{array}$$

(4b) $$\begin{array}{ccc} p & = & \frac{1}{e^{2\nu }}\left(-2\lambda_{tt}-3\lambda_{t}^{2}+2\nu_{t}\lambda_{t}\right)+\frac{1}{e^{2\lambda }}\left(\lambda_{r}^{2}+2\nu_{r}\lambda_{r}+\frac{2\nu_{r}}{r}+\frac{2\lambda_{r}}{r}\right)+\frac{E^{2}}{r^{4}e^{4\lambda }}, \end{array}$$

(4c) $$\begin{array}{ccc} p & = & \frac{1}{e^{2\nu }}\left(-2\lambda_{tt}-3\lambda_{t}^{2}+2\nu_{t}\lambda_{t}\right)+\frac{1}{e^{2\lambda }}\left(\nu_{rr}+\nu_{r}^{2}+\frac{\nu_{r}}{r}+\frac{\lambda_{rr}}{r}+\lambda_{rr}\right)-\frac{E^{2}}{r^{4}e^{4\lambda }}, \end{array}$$

(4d) $$\begin{array}{ccc} 0 & = & \nu_{r}\lambda_{t}-\lambda_{tr}. \end{array}$$

These quantities are measured relative to the comoving fluid 4-velocity $u^{a}=e^{-\nu }\delta_{0}^{a}$. The gravitating equations are supplemented with the Maxwell equations:

(5a) $$\begin{array}{ccc} E & = & r^{2}e^{\lambda -\nu }\Phi_{r}, \end{array}$$

(5b) $$\begin{array}{ccc} E_{r} & = & \sigma r^{2}e^{3\lambda }. \end{array}$$

(The subscripts *r* and *t* represent partial derivatives with respect to *r* and t, respectively.) The term $\Phi_{r}=F_{10}$ is the only nonzero component of the electromagnetic field tensor $F_{ab}=\phi_{b;a}-\phi_{a;b}$ with $\phi_{a}=\left(\Phi \left(t,r\right),0,0,0\right).$ Note that σ is the proper charge density and *E* is the electric field intensity, which represents the total charge of the distribution.

The Einstein–Maxwell system of Equations (4) and (5) can also be written in the equivalent form:

(6a) $$\begin{array}{ccc} \mu & = & 3e^{2h}-e^{-2\lambda }\left(2\lambda_{rr}+\lambda_{r}^{2}+\frac{4\lambda_{r}}{r}\right)-\frac{E^{2}}{r^{4}e^{4\lambda }}, \end{array}$$

(6b) $$\begin{array}{ccc} p & = & \frac{1}{\lambda_{t}e^{3\lambda }}{\left[e^{\lambda }\left(\lambda_{r}^{2}+\frac{2\lambda_{r}}{r}\right)-e^{3\lambda +2h}-\frac{E^{2}}{r^{4}e^{4\lambda }}\right]}_{t}, \end{array}$$

(6c) $$\begin{array}{ccc} e^{\nu } & = & \lambda_{t}e^{-h}, \end{array}$$

(6d) $$\begin{array}{ccc} e^{\lambda }\left(\lambda_{rr}-\lambda_{r}^{2}-\frac{\lambda_{r}}{r}\right) & = & -\rho \left(r\right)-\frac{E^{2}}{r^{4}e^{4\lambda }}, \end{array}$$

(6e) $$\begin{array}{ccc} \sigma & = & r^{-2}e^{-3\lambda }E_{r}, \end{array}$$

where h=h(t) and ρ=ρ(r) are arbitrary functions of integration. The functions *h* and ρ need to be specified in order to find exact solutions for the field equations. The quantity E=E(r) is also a function of integration. The metric function λ is obtained from the condition of pressure isotropy (6d), which has been generalized to include electromagnetic effects. The remaining metric function ν then follows from (6c). The energy density μ and the isotropic pressure *p* can be calculated using Equations (6a) and (6b). Using the transformation:

$$\begin{array}{ccc} x & = & r^{2},\\ y(x,t) & = & e^{-\lambda } \end{array}$$

and setting

$$\begin{array}{ccc} f(x) & = & \frac{\rho }{4r^{2}},\\ g(x) & = & \frac{E^{2}}{2r^{6}}, \end{array}$$

we can rewrite (6d) as:

(7) $$y_{xx}=f\left(x\right)y^{2}+g\left(x\right)y^{3}.$$

The partial differential Equation (7) is the master equation governing the gravitational dynamics of a shear-free charged fluid in general relativity. Since there are no temporal derivatives in (7) we can treat it as an ordinary differential equation but note that the arbitrary quantities that arise from integration are functions of *t*. If the function g=0, then the equation reduces to $y_{xx}=f\left(x\right)y^{2}$ for a neutral fluid. The neutral case has been studied by many researchers including Kustaanheimo [25], Stephani [27], Stephani et al. [28], Maharaj et al. [29], Wafo Soh and Mahomed [40] and Gumede et al. [39].

A recent analysis of the master Equation (7) was performed by Kweyama et al. [21] where they found its first integral by directly integrating this equation using integration by parts. They found the first integral of (7) to be:

(8) $$\begin{array}{c} \tau_{0}\left(t\right)=-y_{x}+f_{I}y^{2}+g_{I}y^{3}-2f_{II}yy_{x}+2f_{III}y_{x}^{2}+2\left[{\left(ff_{II}\right)}_{I}-\frac{1}{3}C_{0}\right]y^{3}+\left[2{\left(gf_{II}\right)}_{I}-C_{1}\right]y^{4}, \end{array}$$

subject to the integrability conditions:

(9a) $$\begin{array}{cc} C_{0} & =2ff_{III}+3{\left(ff_{II}\right)}_{I}+\frac{3}{2}g_{I}, \end{array}$$

(9b) $$\begin{array}{cc} C_{1} & =gf_{III}+2{\left(gf_{II}\right)}_{I}, \end{array}$$

where $C_{0}$ and $C_{1}$ are constants, $\tau_{0}\left(t\right)$ is an arbitrary function of integration, $f_{I}=\int fdx$ and $g_{I}=\int gdx.$ The system (9) is difficult to analyse as they are integral equations. Fortunately, they can be converted to nonlinear differential equations. Solving the integral Equation (9) gives specific forms of f(x) and g(x) in terms of elementary functions. In one instance, these functions are given by:

$$f\left(x\right)=\frac{24}{75}{\left(5b\right)}^{4/5}{\left(x-x_{0}\right)}^{-11/5},$$

and

$$g\left(x\right)=C_{0}{\left(5b\right)}^{-12/5}{\left(x-x_{0}\right)}^{-12/5},$$

where *b* is an arbitrary constant and $x_{0}$ is a constant of integration.

We use a similar approach to obtain a new first integral of the charged generalization (7) subject to different integrability conditions to obtain different forms of f(x) and g(x) in the next section.

## 3. A First Integral

In order to obtain (8), Kweyama et al. [21] adopted a method first suggested by Srivastava [26], and subsequently extended by Maharaj et al. [29]. The approach was simple to apply—the left hand side of (19) was integrated directly and the right hand side integrated by parts. However, note that the difficulty that arises is that the process yields integral equations which need to be solved to complete an exact solution. As a result it is difficult to explicitly find first integrals in practice. There is no algorithm that generates them systematically. Here we show that a new first integral arises, in a simple approach, by adapting a previous method. We multiply Equation (7) by a function to generate a new differential equation, eventually leading to a new first integral. There is no guarantee that this approach will work in general; we find that this simple idea is an effective procedure for a relativistic charged gravitating fluid.

We use a similar technique with one important distinction. We multiply (7) by *x* to obtain:

(10) $$xy_{xx}=\bar{f}y^{2}+\bar{g}y^{3},$$

where for convenience we have let:

$$\bar{f}=xf$$

and

$$\bar{g}=xg.$$

We observe that the left hand side of (10) can still be integrated (by parts), and we can also apply integration by parts to the right hand side. This yields:

(11) $$xy_{x}-y={\bar{f}}_{I}y^{2}+{\bar{g}}_{I}y^{3}-2\int {\bar{f}}_{I}yy_{x}dx-3\int {\bar{g}}_{I}y^{2}y_{x}dx-\psi_{1}\left(t\right),$$

where we have let:

$$\int \bar{f}dx=\int xfdx={\bar{f}}_{I}$$

and

$$\int \bar{g}dx=\int xgdx={\bar{g}}_{I}$$

for convenience, and $\psi_{1}\left(t\right)$ is a function of integration. Integrating ${\bar{f}}_{I}yy_{x}$ and using (7), we obtain:

(12) $$\begin{array}{ccc} xy_{x}-y & = & {\bar{f}}_{I}y^{2}+{\bar{g}}_{I}y^{3}-2{\bar{f}}_{II}yy_{x}+2\int {\bar{f}}_{II}y_{x}^{2}dx+2\int f{\bar{f}}_{II}y^{3}dx\\ & & +2\int g{\bar{f}}_{II}y^{4}dx-3\int {\bar{g}}_{I}y^{2}y_{x}dx-\psi_{1}\left(t\right). \end{array}$$

Integrating $f{\bar{f}}_{II}y^{3}$, $g{\bar{f}}_{II}y^{4}$ and ${\bar{f}}_{II}y_{x}^{2}$ and substituting in (12), we obtain:

(13) $$\begin{array}{ccc} xy_{x}-y & = & {\bar{f}}_{I}y^{2}+{\bar{g}}_{I}y^{3}-2{\bar{f}}_{II}yy_{x}+2{\left(f{\bar{f}}_{II}\right)}_{I}y^{3}+2{\left(g{\bar{f}}_{II}\right)}_{I}y^{4}+2{\bar{f}}_{III}y_{x}^{2}\\ & & -\frac{2}{3}\int \left\{\left[2f{\bar{f}}_{III}+3{\left(f{\bar{f}}_{II}\right)}_{I}+\frac{3}{2}{\bar{g}}_{I}\right]\left(\frac{d(y^{3})}{dx}\right)\right\}dx\\ & & -\int \left\{\left[g{\bar{f}}_{III}+2{\left(g{\bar{f}}_{II}\right)}_{I}\right]\left(\frac{d(y^{4})}{dx}\right)\right\}dx-\psi_{1}\left(t\right). \end{array}$$

The integrals in (13) can be evaluated if:

(14a) $$\begin{array}{cc} K_{0} & =2f{\bar{f}}_{III}+3{\left(f{\bar{f}}_{II}\right)}_{I}+\frac{3}{2}{\bar{g}}_{I}, \end{array}$$

(14b) $$\begin{array}{cc} K_{1} & =g{\bar{f}}_{III}+2{\left(g{\bar{f}}_{II}\right)}_{I}, \end{array}$$

where $K_{0}$ and $K_{1}$ are arbitrary constants. A first integral of (7) is then given by

(15) $$\begin{array}{c} \psi_{1}\left(t\right)=y-xy_{x}+{\bar{f}}_{I}y^{2}+{\bar{g}}_{I}y^{3}-2{\bar{f}}_{II}yy_{x}+2{\bar{f}}_{III}y_{x}^{2}+2\left[{\left(f{\bar{f}}_{II}\right)}_{I}-\frac{1}{3}K_{0}\right]y^{3}+\left[2{\left(g{\bar{f}}_{II}\right)}_{I}-K_{1}\right]y^{4}, \end{array}$$

subject to the integral Equations (14). Note that (15) is a new first integral of (7) subject to new integrability conditions. Thus, the first integral exists for new functions f(x) and g(x) for a charged shear-free matter distribution. We show in Appendix B that this new first integral is independent of the charged first integral found by Kweyama et al. [21].

## 4. Integral Equations

The two equations in (14) are integral equations that need to be solved. To complete the analysis we need to determine the form of the functions f(x) and g(x). In an attempt to seek the form of the functions *f* and g, we rewrite the integral Equations (14) as ordinary differential equations as these are (usually) easier to solve. Setting:

$$\bar{L}={\bar{f}}_{III},$$

and differentiating (14b) we obtain:

$${\left(g\bar{L}\right)}_{x}+2g{\bar{L}}_{x}=0,$$

whose solution is given by:

(16) $$g=K_{2}{\bar{L}}^{-3}.$$

In the equation above, $K_{2}$ is a constant of integration.

Differentiating (14a) and using (16) we obtain:

$$f_{x}\bar{L}+\frac{5}{2}f{\bar{L}}_{x}=-\frac{3}{4}K_{2}x{\bar{L}}^{-3},$$

which can be written as a fourth order differential equation:

(17) $${\left(\frac{1}{x}{\bar{L}}^{5/2}{\bar{L}}_{xxx}\right)}_{x}=-\frac{3}{4}K_{2}x{\bar{L}}^{-3/2},$$

since

$$f=\frac{1}{x}\bar{f}=\frac{1}{x}{\bar{L}}_{xxx}.$$

Integrating (17) three times, we obtain:

(18) $$\begin{array}{c} x^{2}{\bar{L}}^{-1}=K_{6}+\int x{\bar{L}}^{-3/2}dx+\frac{K_{4}}{2}{\left(\int x{\bar{L}}^{-3/2}dx\right)}^{2}+\frac{K_{3}}{2}{\left(\int x{\bar{L}}^{-3/2}dx\right)}^{3}-\frac{K_{2}}{32}{\left(\int x{\bar{L}}^{-3/2}dx\right)}^{4}, \end{array}$$

where $K_{3},K_{4},K_{5}$ and $K_{6}$ are constants of integration. In Appendix A, we illustrate how Equation (17) is integrated repeatedly to yield (18). The solution of (18) can be written parametrically in general. The constant $K_{2}$ is related to the charge. For neutral fluids $K_{2}=0$ and the polynomial in p(u) is third order. For charged fluids $K_{2}\neq 0$ and the polynomial in p(u) is fourth order. Hence, the presence of the electromagnetic field changes the nature of the exact solutions that are permitted when compared to neutral matter.

It is convenient to define:

$$u=\int x{\bar{L}}^{-3/2}dx,$$

so that (18) becomes:

$$x^{2}u_{x}={\left(K_{6}+K_{5}u+\frac{1}{2}K_{4}u^{2}+\frac{1}{6}K_{3}u^{3}-\frac{1}{32}K_{2}u^{4}\right)}^{3/2}.$$

This equation is separable and can be integrated to obtain:

(19) $$x_{0}-\frac{1}{x}=\int \frac{1}{{\left(K_{6}+K_{5}u+\frac{1}{2}K_{4}u^{2}+\frac{1}{6}K_{3}u^{3}-\frac{1}{32}K_{2}u^{4}\right)}^{3/2}},$$

where $x_{0}$ is a constant of integration. The evaluation of the integral on the right hand side of (19) above depends on the nature of the roots of the polynomial $K_{6}+K_{5}u+\frac{1}{2}K_{4}u^{2}+\frac{1}{6}K_{3}u^{3}-\frac{1}{32}K_{2}u^{4}.$ In order to find the functions $\bar{f}\left(x\right)$ and $\bar{g}\left(x\right)$ satisfying the integrability conditions (14), it is convenient to express the solution in the parametric form:

$$\begin{array}{ccc} \bar{f}\left(x\right) & = & {\bar{L}}_{xxx},\\ g & = & K_{2}{\bar{L}}^{-3},\\ u_{x} & = & x{\bar{L}}^{-\frac{3}{2}},\\ x_{0}-\frac{1}{x} & = & p(u), \end{array}$$

where

(20) $$p\left(u\right)=\int \frac{du}{{\left(K_{6}+K_{5}u+\frac{1}{2}K_{4}u^{2}+\frac{1}{6}K_{3}u^{3}-\frac{1}{32}K_{2}u^{4}\right)}^{3/2}}.$$

## 5. Particular Solutions

The evaluation of the integral in (19) can be reduced to nine cases depending on the nature of the factors of the polynomial $K_{6}+K_{5}u+\frac{1}{2}K_{4}u^{2}+\frac{1}{6}K_{3}u^{3}-\frac{1}{32}K_{2}u^{4}$ that appear in p(u). The nine cases correspond to:

**Case I**—One order-four linear factor,
**Case II**—One order-three linear factor,
**Case III**—One order-two linear factor and one order-one quadratic factor,
**Case IV**—One order-two linear factor and two order-one linear factors,
**Case V**—Two order-two linear factors,
**Case VI**—Four non-repeated linear factors,
**Case VII**—One order-two quadratic factor,
**Case VIII**—Two order-one quadratic factors, and
**Case IX**—One order-one cubic factor.

We discuss these cases below.

### 5.1. Case I: One Order-Four Linear Factor

If $K_{6}+K_{5}u+\frac{1}{2}K_{4}u^{2}+\frac{1}{6}K_{3}u^{3}-\frac{1}{32}K_{2}u^{4}$ has one linear factor repeated four times then we have:

$$K_{6}+K_{5}u+\frac{1}{2}K_{4}u^{2}+\frac{1}{6}K_{3}u^{3}-\frac{1}{32}K_{2}u^{4}={\left(a+bu\right)}^{4},$$

with b≠0. The integral in (19) or (20) can be evaluated to obtain:

$$p\left(u\right)=-\frac{1}{5b}{\left(a+bu\right)}^{-5},$$

so that

$$\bar{L}=x^{2}{\left(-\frac{1}{5b}\right)}^{-4/5}{\left(x_{0}-\frac{1}{x}\right)}^{4/5}.$$

Differentiating $\bar{L}$ three times and using (16) we obtain:

(21a) $$\begin{array}{ccc} f(x) & = & \frac{24}{125x^{5}}{\left(-\frac{1}{5b}\right)}^{-4/5}{\left(x_{0}-\frac{1}{x}\right)}^{-11/5}, \end{array}$$

(21b) $$\begin{array}{ccc} g(x) & = & \frac{K_{2}}{x^{6}}{\left(-\frac{1}{5b}\right)}^{12/5}{\left(x_{0}-\frac{1}{x}\right)}^{-12/5}. \end{array}$$

Hence the functions f(x) and g(x) can be found explicitly in this Case I. After reparametrisation we can write:

(22a) $$\begin{array}{ccc} f(x) & ∼ & \frac{1}{x^{5}}{\left(1-\frac{1}{x}\right)}^{-11/5}, \end{array}$$

(22b) $$\begin{array}{ccc} g(x) & ∼ & \frac{1}{x^{6}}{\left(1-\frac{1}{x}\right)}^{-12/5}. \end{array}$$

This is the simplest form. The first integral (15) becomes:

(23) $$\begin{array}{ccc} \psi_{1}\left(t\right) & = & y-xy_{x}+2{\left(-\frac{1}{5b}\right)}^{-4/5}{\left(x_{0}-\frac{1}{x}\right)}^{4/5}y^{2}\\ & & +\frac{8}{5x}{\left(-\frac{1}{5b}\right)}^{-4/5}{\left(x_{0}-\frac{1}{x}\right)}^{-1/5}y^{2}-\frac{4}{25x^{2}}{\left(-\frac{1}{5b}\right)}^{-4/5}{\left(x_{0}-\frac{1}{x}\right)}^{-6/5}y^{2}\\ & & +{\left[\frac{K_{2}}{x^{5}}{\left(-\frac{1}{5b}\right)}^{12/5}{\left(x_{0}-\frac{1}{x}\right)}^{-12/5}\right]}_{I}y^{3}-4x{\left(-\frac{1}{5b}\right)}^{-4/5}{\left(x_{0}-\frac{1}{x}\right)}^{4/5}yy_{x}\\ & & +\frac{8}{5}{\left(-\frac{1}{5b}\right)}^{-4/5}{\left(x_{0}-\frac{1}{x}\right)}^{-1/5}yy_{x}+2x^{2}{\left(-\frac{1}{5b}\right)}^{-4/5}{\left(x_{0}-\frac{1}{x}\right)}^{4/5}y_{x}^{2}\\ & & +2{\left[\frac{48}{125x^{4}}{\left(-\frac{1}{5b}\right)}^{-8/5}{\left(x_{0}-\frac{1}{x}\right)}^{-7/5}+\frac{96}{625x^{2}}{\left(-\frac{1}{5b}\right)}^{-8/5}{\left(x_{0}-\frac{1}{x}\right)}^{-12/5}\right]}_{I}y^{3}\\ & & +2{\left[\frac{2K_{2}}{x^{5}}{\left(-\frac{1}{5b}\right)}^{8/5}{\left(x_{0}-\frac{1}{x}\right)}^{-8/5}+\frac{4K_{2}}{5x^{6}}{\left(-\frac{1}{5b}\right)}^{8/5}{\left(x_{0}-\frac{1}{x}\right)}^{-13/5}\right]}_{I}y^{4}\\ & & -\frac{2}{3}K_{0}y^{3}-K_{1}y^{4}, \end{array}$$

where the subscripts *I* denote the remaining integration. This first integral is a new solution to the Einstein–Maxwell equations for the functions *f* and *g* given in (22). It corresponds to a shear-free spherically symmetric charged fluid. Interestingly, there is no corresponding neutral solution as we must have b≠0 (equivalently $K_{2}\neq 0$) otherwise the polynomial in (20) is not fourth order. This means that charge is always present.

As a final check on our results, we substitute the forms (21a) and (21b) into the integrability conditions (14) in order to find any restrictions on the constants $K_{0}$ and $K_{1}.$ In this case, we find that these constants are both equal to zero. We note that the same restriction occurs in the Kweyama et al. [21] model though this was not observed at that time.

### 5.2. Case II: One Order-Three Linear Factor

If $K_{6}+K_{5}u+\frac{1}{2}K_{4}u^{2}+\frac{1}{6}K_{3}u^{3}-\frac{1}{32}K_{2}u^{4}$ has one order-three linear factor, then we have:

$$K_{6}+K_{5}u+\frac{1}{2}K_{4}u^{2}+\frac{1}{6}K_{3}u^{3}-\frac{1}{32}K_{2}u^{4}=\left(a+bu\right){\left(u+c\right)}^{3}.$$

We can evaluate the integral in (19), with the help of the package Mathematica [41], to obtain:

$$p\left(u\right)=\frac{2\sqrt{\left(a+bu)(u+c\right)}}{35{\left(a-bc\right)}^{5}}\left[\frac{35b^{4}}{a+bu}+\frac{93b^{3}}{u+c}-\frac{29b^{2}\left(a-bc\right)}{{\left(u+c\right)}^{2}}+\frac{13b{\left(a-bc\right)}^{2}}{{\left(u+c\right)}^{3}}-\frac{5{\left(a-bc\right)}^{3}}{{\left(u+c\right)}^{4}}\right].$$

We observe that, in this case, the integral in (19) can be expressed in terms of elementary functions. However, it is not straightforward to perform the inversion to find u(x), and find f(x) and g(x) explicitly as in the previous case.

If we let g=0, $K_{2}=0$ and b=0, then:

$$\begin{array}{ccc} p(u) & = & \frac{2}{7}a^{-3/2}{\left(u+c\right)}^{-7/2},\\ \bar{L} & = & a^{2/7}{\left(\frac{2}{7}\right)}^{-4/2}{\left(-\frac{2}{7}\right)}^{-2/3}x^{2}{\left(x_{0}-\frac{1}{x}\right)}^{6/7}. \end{array}$$

After reparametrisation, f(x) can be written as:

(24) $$f\left(x\right)∼\frac{1}{x^{5}}{\left(1-\frac{1}{x}\right)}^{-15/7},$$

which was found previously in the case of a shear-free spherically symmetric uncharged fluid [39]. The corresponding uncharged first integral is given by:

(25) $$\begin{array}{ccc} \psi_{1}\left(t\right) & = & y-xy_{x}+2{\left(-\frac{2}{7B}\right)}^{-6/7}{\left(x_{0}-\frac{1}{x}\right)}^{6/7}y^{2}+\frac{12}{7x}{\left(-\frac{2}{7B}\right)}^{-6/7}{\left(x_{0}-\frac{1}{x}\right)}^{-1/7}y^{2}\\ & & -\frac{6}{49x^{2}}{\left(-\frac{2}{7B}\right)}^{-6/7}{\left(x_{0}-\frac{1}{x}\right)}^{-8/7}y^{2}-4x{\left(-\frac{2}{7B}\right)}^{-6/7}{\left(x_{0}-\frac{1}{x}\right)}^{6/7}yy_{x}\\ & & -\frac{12}{7}{\left(-\frac{2}{7B}\right)}^{-6/7}{\left(x_{0}-\frac{1}{x}\right)}^{-1/7}yy_{x}+2x^{2}{\left(-\frac{2}{7B}\right)}^{-6/7}{\left(x_{0}-\frac{1}{x}\right)}^{6/7}y_{x}^{2}\\ & & +{\left[\frac{192}{343x^{4}}{\left(-\frac{2}{7B}\right)}^{-12/7}{\left(x_{0}-\frac{1}{x}\right)}^{-9/7}\right]}_{I}y^{3}+{\left[\frac{576}{2401}{\left(-\frac{2}{7B}\right)}^{-12/7}{\left(x_{0}-\frac{1}{x}\right)}^{-16/7}\right]}_{I}y^{3}\\ & & -\frac{2}{3}K_{1}y^{3}, \end{array}$$

as established earlier.

### 5.3. Case III: One Order-Two Linear Factor and One Order-One Quadratic Factor

If $K_{6}+K_{5}u+\frac{1}{2}K_{4}u^{2}+\frac{1}{6}K_{3}u^{3}-\frac{1}{32}K_{2}u^{4}$ has one order-two linear factor and one order-one quadratic factor, then we have:

$$K_{6}+K_{5}u+\frac{1}{2}K_{4}u^{2}+\frac{1}{6}K_{3}u^{3}-\frac{1}{32}K_{2}u^{4}=\left(a+bu+cu^{2}\right){\left(u+d\right)}^{2},$$

with $b^{2}-4ac<0.$ We evaluate the integral in (19) with the aid of Equations (2.266) and (2.269.6) in [42] to obtain:

(26) $$\begin{array}{ccc} p(u) & = & \{\frac{15{\left(b-2cd\right)}^{4}-62c{\left(b-2cd\right)}^{2}\left(a-bd+cd^{2}\right)+24c^{2}{\left(a-bd+cd^{2}\right)}^{2}}{2\left(a-bd+cd^{2}\right)\left[4c\left(a-bd+cd^{2}\right)-{\left(b-2cd\right)}^{2}\right]}\\ \; & \; & +\frac{c\left(b-2cd\right)[15{\left(b-2cd\right)}^{2}-52c\left(a-bd+cd^{2}\right)]u}{2(a-bd+cd^{2})\Delta }\\ \; & \; & -\frac{1}{\left(a-bd+cd^{2}\right)u^{2}}-\frac{5(b-2cd)}{2(a-bd+cd^{2})u}\}\frac{1}{2\sqrt{\left(a-bd+cd^{2}\right)+\left(b-2cd\right)u+cu^{2}}}\\ \; & \; & +\frac{15{\left(b-2cd\right)}^{2}-12c\left(a-bd+cd^{2}\right)}{8{\left(a-bd+cd^{2}\right)}^{3}}\int \frac{du}{u\sqrt{\left(a-bd+cd^{2}\right)+\left(b-2cd\right)u+cu^{2}}}, \end{array}$$

where $\Delta =4\left(a-bd+cd^{2}\right)c-{\left(b-2cd\right)}^{2}.$ The exact form of the integral on the right hand side of (26) depends on the signs of Δ and $a-bd+cd^{2}.$

Specific examples of the constants a,b or *d* make the integral on the right hand side of (19) easier to write in terms of elementary functions. For example, if a=0, then we have

$$K_{6}+K_{5}u+\frac{1}{2}K_{4}u^{2}+\frac{1}{6}K_{3}u^{3}-\frac{1}{32}K_{2}u^{4}=\left(bu+cu^{2}\right){\left(u+d\right)}^{2},$$

which can be evaluated using [42] (Equation (2.269)) to obtain:

$$\begin{array}{ccc} p(u) & = & \frac{2}{7}\{-\frac{1}{\left(b-2cd\right)u^{3}}+\frac{8c}{5\left(b-2cd\right)u^{2}}-\frac{16c^{2}}{5{\left(b-2cd\right)}^{3}u}+\frac{64c^{3}}{5{\left(b-2cd\right)}^{4}}\\ & & +\frac{128c^{4}u}{5{\left(b-2cd\right)}^{5}}\}\frac{1}{\sqrt{\left(b-2cd\right)u+cu^{2}}}. \end{array}$$

As a second example if b=0, then we have:

$$K_{6}+K_{5}u+\frac{1}{2}K_{4}u^{2}+\frac{1}{6}K_{3}u^{3}-\frac{1}{32}K_{2}u^{4}=\left(a+cu^{2}\right){\left(u+d\right)}^{2}.$$

Using Mathematica [41] and evaluating the integral in (19) yields:

$$\begin{array}{ccc} p(u) & = & \frac{1}{2}\{\frac{-a^{3}+2c^{3}d^{3}u{\left(d+u\right)}^{3}-a^{2}c\left(10d^{2}+11du+3u^{2}\right)}{a{\left(a+cd^{2}\right)}^{3}{\left(d+u\right)}^{2}\sqrt{a+cu^{2}}}\\ & & +\frac{ac^{2}d\left(6d^{3}+6d^{2}u-14du^{2}-13u^{3}\right)}{a{\left(a+cd^{2}\right)}^{3}{\left(d+u\right)}^{2}\sqrt{a+cu^{2}}}-\frac{3c\left(a-4cd^{2}\right)\log\left[d+u\right]}{{\left(a+cd^{2}\right)}^{7/2}}\\ & & +\frac{3c\left(a-4cd^{2}\right)\log\left[a-cdu+\sqrt{\left(a+cd^{2}\right)\left(a+cu^{2}\right)}\right]}{{\left(a+cd^{2}\right)}^{7/2}}\}. \end{array}$$

Thirdly, if d=0, then we have that:

$$K_{6}+K_{5}u+\frac{1}{2}K_{4}u^{2}+\frac{1}{6}K_{3}u^{3}-\frac{1}{32}K_{2}u^{4}=\left(a+bu+cu^{2}\right)u^{2}.$$

With the aid of Mathematica [41], we evaluate the integral in (19) to obtain:

$$\begin{array}{ccc} p(u) & = & \frac{1}{8a^{7/2}\left(-b^{2}+4ac\right)u^{2}\sqrt{a+u(b+cu)}}\{-2\sqrt{a}[8a^{3}c+15b^{3}u^{2}\left(b+cu\right)\\ & & +abu\left(5b^{2}-62bcu-52c^{2}u^{2}\right)-2a^{2}\left(b^{2}+10bcu-12c^{2}u^{2}\right)]\\ & & -3\left(5b^{4}-24ab^{2}c+16a^{2}c^{2}\right)u^{2}\sqrt{a+u(b+cu)}\log\left[u\right]\\ & & +3\left(5b^{2}-24ab^{2}c+16a^{2}c^{2}\right)u^{2}\sqrt{a+u(b+cu)}\log\left[2a+bu+2\sqrt{a}\sqrt{a+u(bu+c)}\right]\}, \end{array}$$

expressed in terms of elementary functions. As a fourth example, if d=b=0, then (19) becomes:

$$p\left(u\right)=\frac{-\sqrt{a}\left(a+3cu^{2}\right)-3cu^{2}\sqrt{a+cu^{2}}\left(\log\left[u\right]-\log\left[a+\sqrt{a}\sqrt{a+cu^{2}}\right]\right)}{2a^{5/2}u^{2}\sqrt{a+cu^{2}}}.$$

Finally, we study the case where a=d=0. In this case, evaluating the integral in (19) yields

$$p\left(u\right)=\frac{2(-5b^{4}+8b^{3}cu-16b^{2}c^{2}u^{2}+64bc^{3}u^{3}+128c^{4}u^{4})}{35b^{5}u^{3}\sqrt{u(b+cu)}}.$$

We observe that if the polynomial $K_{6}+K_{5}u+\frac{1}{2}K_{4}u^{2}+\frac{1}{6}K_{3}u^{3}-\frac{1}{32}K_{2}u^{4}$ has one order-two linear factor and one order-one quadratic factor, it is difficult to obtain f(x) and g(x) explicitly as the expressions for *p* cannot be inverted to obtain u.

### 5.4. Case IV: One Order-Two Linear Factor and Two Order-One Linear Factors

If the polynomial $K_{6}+K_{5}u+\frac{1}{2}K_{4}u^{2}+\frac{1}{6}K_{3}u^{3}-\frac{1}{32}K_{2}u^{4}$ has one order-two linear factor and two order-one linear factors, then we have:

$$K_{6}+K_{5}u+\frac{1}{2}K_{4}u^{2}+\frac{1}{6}K_{3}u^{3}-\frac{1}{32}K_{2}u^{4}=\left(a+bu\right)\left(c+du\right){\left(u+e\right)}^{2}.$$

In this case, the integral in (19) can be expressed completely in terms of elementary functions and can be obtained using Mathematica [41]. However, we do not include it here due to its length, and the fact that *u* cannot be obtained explicitly.

### 5.5. Case V: Two Order-Two Linear Factors

With two order-one linear factors, we have:

$$K_{6}+K_{5}u+\frac{1}{2}K_{4}u^{2}+\frac{1}{6}K_{3}u^{3}-\frac{1}{32}K_{2}u^{4}={\left(a+bu\right)}^{2}{\left(u+c\right)}^{2}.$$

The integral in (19) may be evaluated to obtain:

$$\begin{array}{ccc} p(u) & = & \frac{3b^{2}}{{\left(a-bc\right)}^{4}\left(a+bu\right)}+\frac{6b^{2}\log\left[u+c\right]}{{\left(a-bc\right)}^{5}}+\frac{1}{2{\left(a-bc\right)}^{3}{\left(a+bu\right)}^{2}}\\ & & -\frac{6b^{2}\log\left[a+u\right]}{{\left(a-bc\right)}^{5}}-\frac{1}{2{\left(a-bc\right)}^{3}{\left(u+c\right)}^{2}}+\frac{3b}{{\left(a-bc\right)}^{4}\left(u+c\right)}. \end{array}$$

Setting the constants a=0 or c=0 simplifies the result. For example, if c=0, then we have:

$$\begin{array}{ccc} p(u) & = & \frac{3b^{2}}{a^{4}\left(a+bu\right)}+\frac{6b^{2}\log\left[u\right]}{a^{5}}+\frac{b^{2}}{2a^{3}{\left(a+bu\right)}^{2}}\\ & & -\frac{6b^{2}\log\left[a+bu\right]}{a^{5}}+\frac{3b}{a^{4}u}-\frac{1}{2a^{3}u^{2}}, \end{array}$$

while for a=0 we have [43]:

$$\begin{array}{ccc} p(u) & = & \frac{6\log[u]}{b^{3}c^{5}}-\frac{6\log[u+c]}{b^{3}c^{5}}+\frac{3}{b^{3}c^{4}u}+\frac{1}{2b^{3}c^{3}{\left(u+c\right)}^{2}}\\ & & +\frac{3}{b^{3}c^{4}\left(u+c\right)}-\frac{1}{2b^{3}c^{3}u^{2}}. \end{array}$$

However, due to the combination of logarithmic terms and powers of *u*, one cannot invert in order to obtain u(x) explicitly.

### 5.6. Case VI: Four Non-Repeated Linear Factors

With four non-repeated linear factors, we have:

$$K_{6}+K_{5}u+\frac{1}{2}K_{4}u^{2}+\frac{1}{6}K_{3}u^{3}-\frac{1}{32}K_{2}u^{4}=e\left(a+u\right)\left(b+u\right)\left(c+u\right)\left(d+u\right),$$

with e≠0. The integral in (19) is given by:

(27) $$\begin{array}{ccc} p(u) & = & \frac{2e^{-3/2}}{\left(a-b\right)\sqrt{\left(a+u)(b+u)(c+u)(d+u\right)}}\\ \; & \; & \times \left[\frac{\left(a+u)(b+u\right)}{\left(b-c)(a-d\right)}\right]\left[\frac{2}{{\left(b-d\right)}^{2}}\frac{1}{\left(b-d)(c-d\right)}+\frac{1}{\left(a-c)(c-d\right)}\right]\\ \; & \; & +\left[\frac{\left(a+u)(b+u\right)}{\left(b-c)(a-d\right)}\right]\left[\left[\frac{2(d+u)(b+u)}{\left(a-b\right){\left(a-d\right)}^{2}}\frac{1}{\left(b-c)(b-d)(a-c\right)}\right]-\frac{b+u}{\left(b-c)(b-d)(a-c\right)}\right]\\ \; & \; & -\frac{4e^{-3/2}}{\left(a-b\right)\sqrt{b-d}}\left[\frac{1}{{\left(a-d\right)}^{2}\left(c-d\right)\sqrt{a-c}}+\frac{\sqrt{a-c}}{\left(a-b\right)\left(b-d\right){\left(b-c\right)}^{2}}\right]E\left(\alpha ,p\right)\\ & \; & -\frac{4e^{-3/2}}{\left(a-b\right)\sqrt{b-d}}\left[\frac{a-b-c+d}{\left(b-c\right){\left(c-d\right)}^{2}{\left(a-c\right)}^{3/2}}\right]E\left(\alpha ,p\right)\\ \; & \; & +\frac{2e^{-3/2}}{\left(a-d\right)\left(b-c\right){\left(a-c\right)}^{3/2}{\left(b-d\right)}^{3/2}}\times \left[\frac{2{\left(a+b-c-d\right)}^{2}}{\left(b-c)(a-d\right)}\right]F\left(\alpha ,p\right)\\ \; & \; & +\left[\frac{{\left(a-b-c+d\right)}^{2}}{\left(a-b)(c-d\right)}\right]F\left(\alpha ,p\right),\;\left(0<d<c<b<a\right). \end{array}$$

In the above

$$\alpha =\arcsin\sqrt{\frac{\left(a-c)(u+d\right)}{\left(a-d)(u+c\right)}},\;p=\frac{\left(b-c)(a-d\right)}{\left(a-c)(b-d\right)},$$

and F(α,p) and E(α,p) are the elliptic integrals of the first and second kind, respectively (See also [21]). We did not include the special cases in this case because they do not simplify the integral, which are still in terms of elliptic integrals and they do not have an uncharged limit.

### 5.7. Case VII: One Order-Two Quadratic Factor

With one order-two quadratic factor, we have:

$$K_{6}+K_{5}u+\frac{1}{2}K_{4}u^{2}+\frac{1}{6}K_{3}u^{3}-\frac{1}{32}K_{2}u^{4}={\left(a+bu+cu^{2}\right)}^{2}.$$

Using Equation (2.173.2) in [42], the integral in (19) may be evaluated to obtain:

$$p\left(u\right)=\frac{b+2cu}{4ac-b^{2}}\left[\frac{1}{2{\left(a+bu+cu^{2}\right)}^{2}}+\frac{3c}{\left(4ac-b^{2}\right)\left(a+bu+cu^{2}\right)}\right]+\frac{6c^{2}}{{\left(4ac-b^{2}\right)}^{2}}\int \frac{du}{a+bu+cu^{2}},$$

where the integral on the right hand side depends on the sign of $4ac-b^{2}.$ In the special case of a=0 we have:

$$K_{6}+K_{5}u+\frac{1}{2}K_{4}u^{2}+\frac{1}{6}K_{3}u^{3}-\frac{1}{32}K_{2}u^{4}={\left(bu+cu^{2}\right)}^{2},$$

so that:

$$p\left(u\right)=\frac{4b^{2}cu-b^{3}+18bc^{2}u^{2}+12c^{3}u^{3}}{2b^{4}u^{2}{\left(b+cu\right)}^{2}}+6c^{2}\frac{\log[u]-\log[b+cu]}{b^{5}}.$$

For b=0 we have:

$$K_{6}+K_{5}u+\frac{1}{2}K_{4}u^{2}+\frac{1}{6}K_{3}u^{3}-\frac{1}{32}K_{2}u^{4}={\left(a+cu^{2}\right)}^{2},$$

which yields:

$$p\left(u\right)=\frac{5au+3cu^{3}}{8a^{2}{\left(a+cu^{2}\right)}^{2}}+\frac{3\arctan[\frac{u\sqrt{c}}{\sqrt{a}}]}{8a^{5/2}\sqrt{c}}.$$

### 5.8. Cases VIII and IX

In cases VIII and IX, the integral in (19) can also be evaluated using Mathematica [41]. The solution can be expressed in terms of elementary functions as well as elliptic integrals but are omitted due to space considerations.

We therefore conclude that it is only in the case where $K_{6}+K_{5}u+\frac{1}{2}K_{4}u^{2}+\frac{1}{6}K_{3}u^{3}-\frac{1}{32}K_{2}u^{4}$ has one linear factor repeated four times that we can easily use the integral in (19) to obtain specific functional forms of f(x) and g(x) that satisfy the integrability conditions (14).

## 6. Discussion

In this paper, we studied the equation $y_{xx}=f\left(x\right)y^{2}+g\left(x\right)y^{3}$ which is a charged generalization of the Emden–Fowler equation. This equation is a consequence of the Einstein–Maxwell system of field equations, and it is important for describing the evolution of a relativistic charged shear-free matter distribution. We multiplied the charged version of the Emden–Fowler equation by an integrating factor and obtained a new first integral (15), which is subject to consistency conditions (14). We emphasize that the conditions (14) are integral equations. Note that earlier charged first integrals are not contained in this solution. In particular we do not regain the result of Kweyama et al. [21]. Thus, our results complement existing treatments and provide an independent analysis of the charged Emden–Fowler Equation (7). Therefore, charged shear-free fluids display desirable features of complexity in our treatment.

We summarize the results that have been obtained for Equation (7) in terms of first integrals. For neutral matter with g(x)=0, particular results were obtained by Stephani [27], Srivastava [26], Maharaj et al. [29], Wafo Soh and Mahomed [40] and Gumede et al. [39]. Some simple forms of the function f(x) that were identified correspond to

$$f\left(x\right)∼x^{-15/7},$$

and

$$f\left(x\right)∼\frac{1}{x^{5}}{\left(1-\frac{1}{x}\right)}^{-15/7}.$$

For charged matter with g(x)≠0 first integrals were obtained by Kweyama et al. [21] and the results contained in this paper. The functional forms of f(x) and g(x) are given by:

$$f\left(x\right)∼{\left(1-\frac{1}{x}\right)}^{-11/5},\;g\left(x\right)∼{\left(1-\frac{1}{x}\right)}^{-12/5},$$

and

$$f\left(x\right)∼\frac{1}{x^{5}}{\left(1-\frac{1}{x}\right)}^{-11/5},\;g\left(x\right)∼\frac{1}{x^{6}}{\left(1-\frac{1}{x}\right)}^{-12/5}.$$

The charged solutions arise as repeated roots of a fourth order polynomial. Note that the charged models do not have an uncharged limit since the polynomial then becomes a cubic, which is a contradiction. Our results indicate that the complexity of the system is affected by the presence of the electromagnetic field. In future work it would be interesting to investigate complexity in general dissipative fluids, including electromagnetic effects, and to consider geometries with less symmetry such as cylindrical and axial spacetimes.

## Data Availability

This manuscript has no associated data.

## Appendix A. Integration of (17)

In this appendix we illustrate how the Equation (17) is integrated to yield (18). Integrating (17) once we obtain

$$\frac{1}{x}{\bar{L}}^{5/2}{\bar{L}}_{xxx}=K_{3}-\frac{3}{4}K_{2}\int x{\bar{L}}^{-3/2}dx.$$

Multiplying this equation by $x{\bar{L}}^{-3/2}$ and writing the left hand side as a total derivative we obtain

$${\left(\bar{L}{\bar{L}}_{xx}\right)}_{x}-\frac{1}{2}{\left({\bar{L}}_{x}^{2}\right)}_{x}=K_{3}x{\bar{L}}^{-3/2}-\frac{3}{4}K_{2}x{\bar{L}}^{-3/2}\int x{\bar{L}}^{-3/2}dx,$$

which integrates to

$$\bar{L}{\bar{L}}_{xx}-\frac{1}{2}{\bar{L}}_{x}^{2}=K_{4}+K_{3}\int x{\bar{L}}^{-3/2}dx-\frac{3}{8}K_{2}{\left(\int x{\bar{L}}^{-3/2}\right)}^{2}.$$

Multiplying this equation by $x{\bar{L}}^{-3/2}$ we can rewrite it as

(A1) $$\begin{array}{ccc} x{\left({\bar{L}}^{1/2}\right)}_{xx} & = & K_{4}x{\bar{L}}^{-3/2}+K_{3}x{\bar{L}}^{-3/2}\int x{\bar{L}}^{-3/2}dx\\ & & -\frac{3}{8}K_{2}x{\bar{L}}^{-3/2}{\left(\int x{\bar{L}}^{-3/2}\right)}^{2}. \end{array}$$

Since

$$x{\left({\bar{L}}^{1/2}\right)}_{xx}={\left[x{\left({\bar{L}}^{1/2}\right)}_{x}\right]}_{x}-{\left({\bar{L}}^{1/2}\right)}_{x},$$

Equation (A1) can be written as

$${\left[x{\left({\bar{L}}^{1/2}\right)}_{x}\right]}_{x}-{\left({\bar{L}}^{1/2}\right)}_{x}=K_{4}x{\bar{L}}^{-3/2}+K_{3}x{\bar{L}}^{-3/2}\int x{\bar{L}}^{-3/2}dx-\frac{3}{8}K_{2}x{\bar{L}}^{-3/2}{\left(\int x{\bar{L}}^{-3/2}\right)}^{2},$$

and integrated to obtain

$${\left({\bar{L}}^{1/2}\right)}_{x}-{\bar{L}}^{1/2}=K_{4}x{\bar{L}}^{-3/2}+K_{3}x{\bar{L}}^{-3/2}\int x{\bar{L}}^{-3/2}dx-\frac{3}{8}K_{2}x{\bar{L}}^{-3/2}{\left(\int x{\bar{L}}^{-3/2}\right)}^{2}.$$

Multiplying the equation above by $x{\bar{L}}^{-3/2}$ and writing the left hand side as a total derivative we obtain

$${\left(-\frac{1}{2}x^{2}{\bar{L}}^{-1}\right)}_{x}=K_{4}x{\bar{L}}^{-3/2}+K_{3}x{\bar{L}}^{-3/2}\int x{\bar{L}}^{-3/2}dx-\frac{3}{8}K_{2}x{\bar{L}}^{-3/2}{\left(\int x{\bar{L}}^{-3/2}\right)}^{2}.$$

Integrating yields

$$\begin{array}{ccc} x^{2}{\bar{L}}^{-1} & = & K_{6}+K_{5}\int x{\bar{L}}^{-3/2}dx+\frac{K_{4}}{2}{\left(\int x{\bar{L}}^{-3/2}\right)}^{2}+\frac{K_{3}}{6}{\left(\int x{\bar{L}}^{-3/2}\right)}^{3}\\ & & -\frac{K_{2}}{32}{\left(\int x{\bar{L}}^{-3/2}\right)}^{4}, \end{array}$$

where we absorb a factor of $-\frac{1}{2}$ into the $K_{i}$’s.

## Appendix B. Independence of the Result (15)

In this Appendix, we explore the possibility of both our first integral (15) and that of Kweyama et al. [21] existing simultaneously. We note that those two first integrals exist subject to the integrability conditions (14) and (9). Differentiating Equations (9b) and (14b) leads to

(A2a) $$\begin{array}{ccc} 2gf_{II}+{\left(gf_{III}\right)}_{x} & = & 0 \end{array}$$

(A2b) $$\begin{array}{ccc} 2g{\bar{f}}_{II}+{\left(g{\bar{f}}_{III}\right)}_{x} & = & 0. \end{array}$$

The general solution of (A2a) is given by

(A3) $$g=K_{4}{\left(f_{III}\right)}^{-3}$$

Now, if we substitute (A3) into (A2b) we obtain the fourth order integral equation

(A4) $$3f_{IIII}f_{II}-2{\left(f_{III}\right)}^{2}=0,$$

whose solution is

(A5) $$f_{IIII}=\frac{1}{27}{\left(K_{7}x+K_{8}\right)}^{3}.$$

Differentiating $f_{IIII}$ four times leads to f=0. In order to find the form of *g* that corresponds to f=0, we substitute f=0 in (7) to obtain

(A6) $$y_{xx}=gy^{3},$$

whose first integral is given by

(A7) $$\begin{array}{ccc} y_{x} & = & g_{I}y^{3}-3\int g_{I}y^{2}y_{x}dx \end{array}$$

(A8) $$\begin{array}{ccc} & = & g_{I}y^{3}-3\int g_{I}\frac{1}{3}\left(\frac{dy^{3}}{dx}\right). \end{array}$$

The integral on the right hand side of (A7) can be evaluated if $g_{I}=\bar{C_{0}},$ hence g(x)=0. Similarly, if we let f=0 in (10), the resulting first integral can be evaluated if ${\bar{g}}_{I}=\bar{C_{1}};$ that is if g=0 as before.

Thus, the requirements of both sets of integrability conditions, arising from (9) and (14), force f=g=0. This implies that the first integrals (8) and (15) are independent of each other.
